# Supplementary material for: PCSK9 Confers Inflammatory Properties to Extracellular Vesicles Released by Vascular Smooth Muscle Cells
Source: Int J Mol Sci. 2022 Oct 28;23(21):13065. doi: 10.3390/ijms232113065 (PMC9655172; doi:10.3390/ijms232113065)
Supplement: Supplementary file 1 [file ijms-23-13065-s001.zip › ijms-1987961-supplementary.pdf]

Supplemental Figure S1

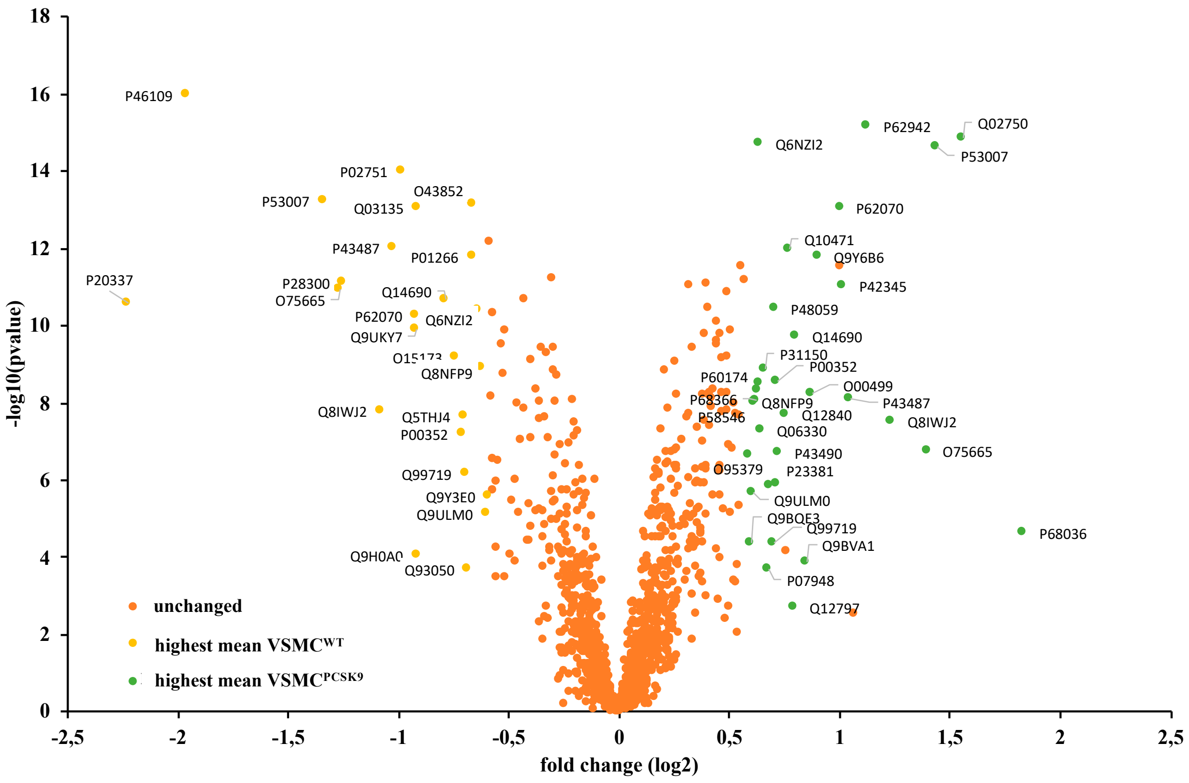

Supplemental Figure S1. Volcano plot of proteins mostly expressed in vascular smooth muscle cells overexpressing PCSK9. PCSK9, proprotein convertase subtilisin/kexin type 9.

Supplemental Figure S2

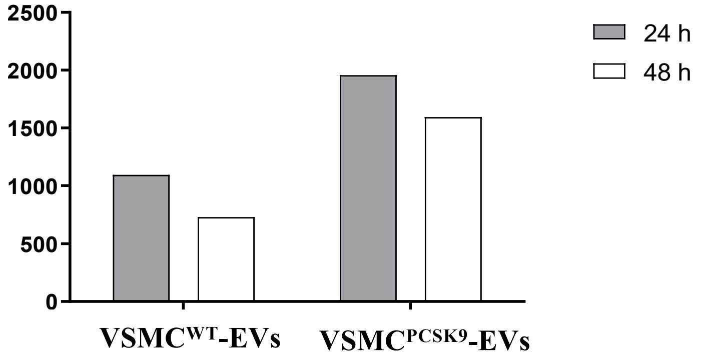

Supplemental Figure S2. Time course experiment to assess optimal conditions for EVs isolation. VSMCs were maintained in DMEM. EVs were isolated from the conditioned medium after 24 and 48 hours and then the concentration was assessed by NTA. NTA, nanoparticle tracking analysis; VSMC, vascular smooth muscle cells.

Supplemental Figure S3

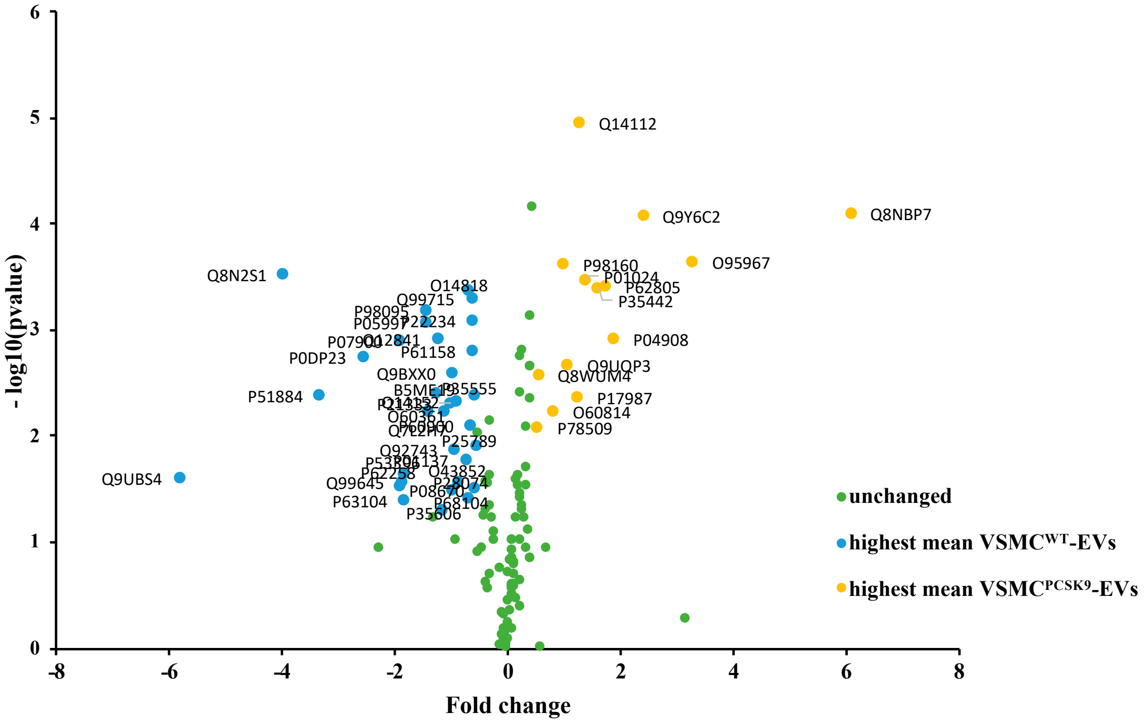

Supplemental Figure S3. Volcano plot of proteins mostly expressed in extracellular vesicles (EVs) released from vascular smooth muscle cells overexpressing PCSK9. PCSK9, proprotein convertase subtilisin/kexin type 9.

Supplemental Figure S4

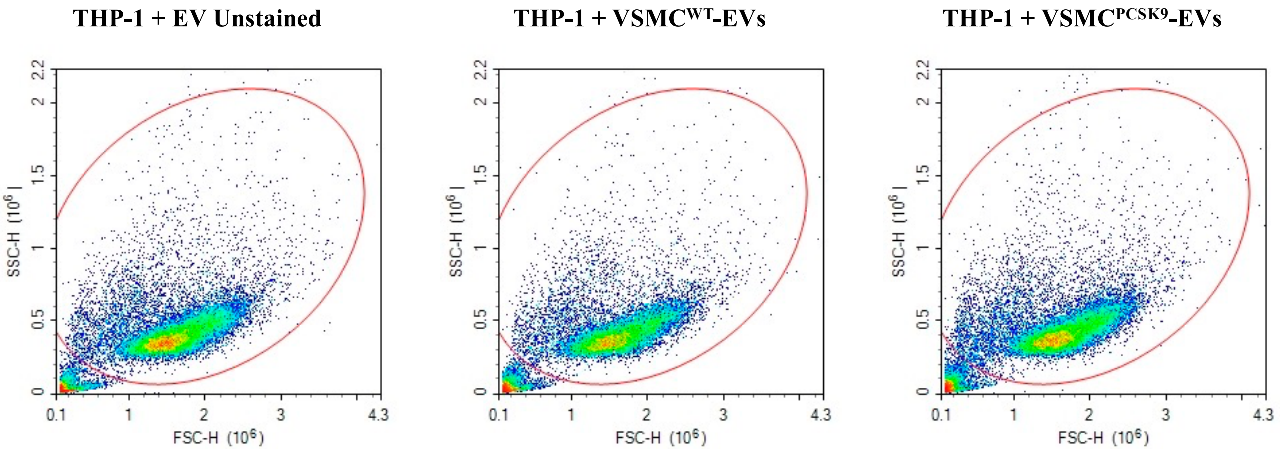

Supplemental Figure S4. Dot plot relative to the uptake of VSMCPCSK9-EVs and VSMCWT-EVs by target cells (THP-1) assessed by flow cytometry. VSMC^{PCSK9}-EVs, EVs released by VSMCs overexpressing PCSK9; VSMC^{WT}-EVs, EVs released by VSMCs wildtype

Supplemental Figure S5

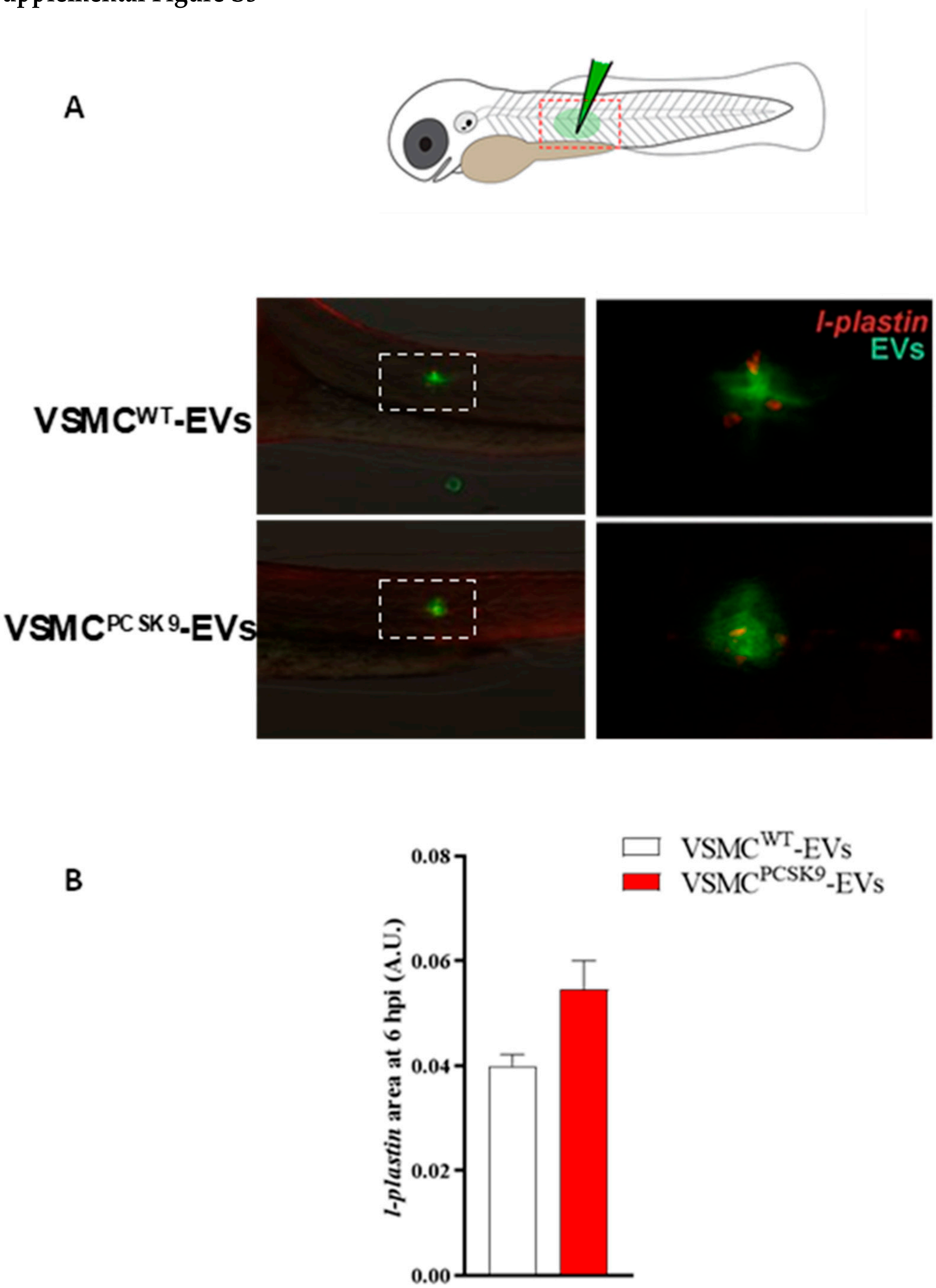

Supplemental Figure S5. Local microinjection of EVs into embryos of zebrafish. A) Immuno-detection assay (anti-l-plastin) in trunk area (lateral view) of embryos treated with CFSE-stained EVs. A scheme of injection strategy is reported. Representative fluorescence overlaid with brightfield images shows macrophage (red) and EV (green) recruitment at the injection site (dashed line) 6 hpi. B) Macrophage quantification (l-plastin positive signal area) in dashed line area at 6 hpi; the mean values of l-plastin positive signal area per embryo ± SEM are indicated (VSMC^{WT}-EVs= 20 and VSMC^{PCSK9}-EVs= 16, from three independent biological replicates). Red bar represents the injection of VSMC^{PCSK9}-EVs; white bar represents the injection with VSMC^{WT}-EVs. Statistical significance was assessed by two-tailed unpaired Student's t test; scale bar 300 μm. A.U., arbitrary unit; EVs, extracellular vesicles; hpi, hours post injection; SEM, standard error of the mean; VSMC^{PCSK9}-EVs, EVs released by VSMCs overexpressing PCSK9; VSMC^{WT}-EVs, EVs released by VSMCs wildtype.

Supplemental Figure S6

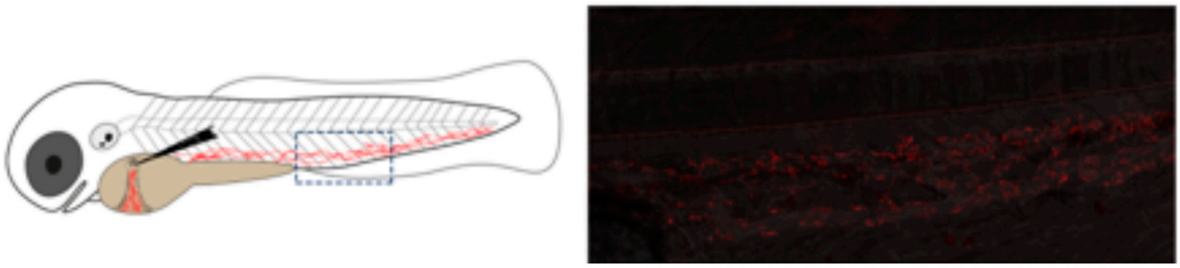

Supplemental Figure S6. Biodistribution of EVs stained with sulfo-cyanine 7.5 (red) systematically injected into the caudal region of zebrafish embryos. EVs, extracellular vesicles.

Supplemental Table S1. List of primer sequences used for qRT-PCR analysis

	Forward(5'-3')	*Reverse(5'-3')*
	**Cell culture**	
**IL-1α**	GTAGCAACCAACGGGAAGGT	AAGGTGCTGACCTAGGCTTG
**IL-1β**	TACCTGTCCTGCGTGTTGAA	TCTTTGGGTAATTTTTGGGATCT
**IL-6**	ACCCCCAGGAGAAGATTCCA	GGTTGTTTTCTGCCAGTGCC
**CCL2**	AGCCACCTTCATTCCCCAAG	CTCCTTGGCCACAATGGTCT
**IL-8**	GAGCACTCCATAAGGCACAAA	ATGGTTCCTTCCGGTGGT
**ET-1**	AGAGTGTGTCTACTTCTGCCA	CTTCCAAGTCCATACGGAACAA
**ICAM-1**	CTCCAATGTGCCAGGCTTG	CAGTGGGAAAGTGCCATCCT
**VCAM-1**	TTCCCTAGAGATCCAGAAATCGAG	CTTGCAGCTTACAGTGACAGAGC
**18S**	CGGCTACCACATCCAAGGAA	CCTGTATTGTTATTTTTCGTCACTACCT
**36B4**	CCACGCTGCTGAACATGC	TCGAACACCTGCTGATGAC
	**Zebrafish**	
**Rpl8**	CTCCGTCTTCAAAGCCCATGT	TCCTTCACGATCCCCTTGATG
**β-actin**	GCACGAGAGATCTTCACTCC	GCAGCGATTTCCTCATCCAT
**IL-16**	TGGACTTCGCAGCACAAAATG	CGTTCACTTCACGCTCTTGGATG
**CXCL8**	CGACGCATTGGAAAACACAT	TGTCATCAAGGTGGCAATGA

IL-1α= Interleukin-1α; IL-1β= Interleukin-1β; IL-6= Interleukin-6; CCL2= C-C motif chemokine ligand 2; IL-8= Interleukin-8; ET-1= Endothelin 1; ICAM-1= Intercellular adhesion molecule 1; VCAM-1= Vascular cell adhesion molecule 1; Rpl8= 60S ribosomal protein L8; IL-16= Interleukin-16; CXCL8= C-X-C Motif Chemokine Ligand 8.

Supplemental Table S2. List of primary antibodies used for western blot and immunofluorescence analyses

Primary antibodies	***Dilution***
Alix (Abcam, Cambridge, UK)	1:1000
β1-Integrin (Abcam, Cambridge, UK)	1:1000
β-Actin (Santa Cruz Biotechnology, Santa Cruz, CA)	1:1000
CD36 (Proteintech, Manchester, UK)	1:2000
PCSK9 (Genetex, Irvine, CA)	1:1000
pSTAT3 (Abcam, Cambridge, UK)	1:10,000
SOCS3 (Proteintech, Manchester, UK)	1:1000
Vinculin (Genetex, Irvine, CA)	1:1000
LCP1 (Genetex, Irvine, CA)	1:200

CD36= Cluster of differentiation 36; PCSK9= Proprotein convertase subtilisin/kexin type 9; pSTAT3= phospho-Signal Transducer And Activator Of Transcription 3; SOCS3= Suppressor of Cytokine Signaling 3; LCP1= L-Plastin.

Supplemental Table S3. Gene Ontology (GO) enrichment analysis performed on the 33 proteins with the highest expression in VSMCs^{PCSK9}

GO:term	*Description*	*FDR*
GO:0006096	Glycolytic process	0.00023
GO:0019674	NAD metabolic process	0.00023
GO:0032386	Regulation of intracellular transport	0.00032
GO:0061621	Canonical glycolysis	0.00032
GO:0006091	Generation of precursor metabolites and energy	0.00043
GO:0055114	Oxidation-reduction process	0.00043
GO:0051640	Organelle localization	0.00064
GO:0006094	Gluconeogenesis	0.0012
GO:0046034	ATP metabolic process	0.0012
GO:0016051	Carbohydrate biosynthetic process	0.0017
GO:0051656	Establishment of organelle localization	0.0026
GO:0051128	Regulation of cellular component organization	0.0039
GO:0010770	Positive regulation of cell morphogenesis involved in differentiation	0.0046
GO:0060341	Regulation of cellular localization	0.0046
GO:0022603	Regulation of anatomical structure morphogenesis	0.0076
GO:0051234	Establishment of localization	0.0083
GO:0051179	Localization	0.017
GO:0090170	Regulation of Golgi inheritance	0.017
GO:0099637	Neurotransmitter receptor transport	0.017
GO:0048489	Synaptic vesicle transport	0.0186
GO:0006810	Transport	0.0194
GO:0010720	Positive regulation of cell development	0.0194
GO:0045597	Positive regulation of cell differentiation	0.0194
GO:0048699	Generation of neurons	0.021
GO:0032502	Developmental process	0.0277
GO:0009117	Nucleotide metabolic process	0.0313
GO:0016185	Synaptic vesicle budding from presynaptic endocytic zone membrane	0.0313
GO:0050793	Regulation of developmental process	0.0348
GO:0019682	glyceraldehyde-3-phosphate metabolic process	0.0354
GO:0098884	Postsynaptic neurotransmitter receptor internalization	0.0354
GO:0030388	Fructose 1,6-bisphosphate metabolic process	0.041
GO:0007399	Nervous system development	0.0424
GO:0051649	Establishment of localization in cell	0.0424
GO:0005975	Carbohydrate metabolic process	0.0437
GO:0032879	Regulation of localization	0.0437
GO:0050790	Regulation of catalytic activity	0.0437
GO:1902959	Regulation of aspartic-type endopeptidase activity involved in amyloid precursor protein catabolic process	0.0437
GO:0042221	Response to chemical	0.0447
GO:0051049	Regulation of transport	0.0471
GO:0071496	Cellular response to external stimulus	0.0477
GO:1903800	Positive regulation of production of miRNAs involved in gene silencing by miRNA	0.0477

GO:0051130	Positive regulation of cellular component organization	0.0495
GO:1903034	Regulation of response to wounding	0.0495
GO:0016192	Vesicle-mediated transport	0.0498
GO:0022604	Regulation of cell morphogenesis	0.0498

Supplemental Table S4. Gene Ontology (GO) enrichment analysis on the 14 proteins with the highest expression in EVs^{PCSK9}.

GO term ID	*Description*	*FDR < 0.05*
GO:0005201	Extracellular matrix structural constituent	0.0038
GO:0070325	Lipoprotein particle receptor binding	0.0038
GO:0070326	Very-low-density lipoprotein particle receptor binding	0.0077
GO:0005102	Signaling receptor binding	0.0380

Supplemental Table S5. Gene targets associated with inflammation and atherosclerosis.

Gene	*miRNA*
ACKR1	hsa-miR-34c-5p
ADIPOR1	hsa-miR-221-3p
ADIPOR2	hsa-miR-34c-5p
AGTR1	hsa-miR-34c-5p
ALOX5	hsa-miR-29a-5p
ANXA1	hsa-miR-221-3p
APLN	hsa-miR-497-5p
APP	hsa-miR-497-5p
BDNF	hsa-miR-497-5p
CCR7	hsa-miR-125b-5p
CD163	hsa-miR-497-5p
CD28	hsa-miR-497-5p
CD40	hsa-miR-497-5p
CD47	hsa-miR-497-5p, hsa-miR-34c-5p
CNR1	hsa-miR-221-3p
CSF1	hsa-miR-497-5p
CTNNB1	hsa-miR-148b-5p
CTSB	hsa-miR-34c-5p
CX3CL1	hsa-miR-497-5p
CXCL12	hsa-miR-221-3p
CXCL16	hsa-miR-34c-5p
DLL4	hsa-miR-497-5p
EDN1	hsa-miR-125b-5p
EFNB1	hsa-miR-34c-5p
EPO	hsa-miR-125b-5p
FCGR3A	hsa-miR-148b-5p
FGF1	hsa-miR-497-5p
FGF2	hsa-miR-497-5p
FOXP3	hsa-miR-34c-5p
IFNG	hsa-miR-125b-5p
IL13	hsa-miR-125b-5p
IL15	hsa-miR-148b-5p, hsa-miR-497-5p
IL20	hsa-miR-497-5p
LIF	hsa-miR-125b-5p
MAPK14	hsa-miR-125b-5p
NRG1	hsa-miR-221-3p
PCSK1	hsa-miR-29a-5p
PDGFA	hsa-miR-221-3p
PGF	hsa-miR-34c-5p
PLA2G6	hsa-miR-34c-5p
PTEN	hsa-miR-497-5p
PTPN1	hsa-miR-125b-5p

SAA1	hsa-miR-125b-5p
SERPINE1	hsa-miR-34c-5p
SIRT1	hsa-miR-34c-5p
SMAD3	hsa-miR-497-5p
SMAD7	hsa-miR-497-5p
STAT3	hsa-miR-125b-5p
TFRC	hsa-miR-497-5p
THBD	hsa-miR-34c-5p
TNFSF13B	hsa-miR-497-5p
TP73	hsa-miR-125b-5p
VEGFA	hsa-miR-497-5p
WDR1	hsa-miR-125b-5p

Supplemental Table S6. List of differentially expressed proteins in the secretome of THP-1 exposed to EVs^{PCSK9}

Accession	*Description*	*Peptide count*	*Unique peptides*	*Confidence score*	*p value*	*Max fold change*
P50552	Vasodilator-stimulated phosphoprotein	7	6	44.2	8.76E-07	2.00
P10145	Interleukin-8	4	4	48.8	6.19E-04	1.64
P09972	Fructose-bisphosphate aldolase C	9	4	108.1	1.08E-05	1.54
O94804	Serine/threonine-protein kinase 10	3	2	16.8	1.88E-02	1.54
P09341	Growth-regulated alpha protein	4	3	30.9	2.04E-03	1.54
P29966	Myristoylated alanine-rich C-kinase substrate	12	10	102.4	6.43E-03	1.52
Q9H3K6	BolA-like protein 2	2	2	14.4	5.25E-04	1.47
P30405	Peptidyl-prolyl cis-trans isomerase F mitochondrial	4	2	29.4	2.00E-04	1.46
P04083	Annexin A1	5	5	43.4	2.90E-03	1.46
O00764	Pyridoxal kinase	5	5	50.2	3.67E-03	1.36
P61077	Ubiquitin-conjugating enzyme E2 D3	3	3	21.3	4.93E-03	1.32
Q15942	Zyxin	5	5	34.2	7.03E-04	1.31

P51991	Heterogeneous nuclear ribonucleoprotein A3	10	6	85.6	1.05E-04	1.30
Q15366	Poly(rC)-binding protein 2	4	2	29.4	1.15E-03	1.29
Q9BWD1	Acetyl-CoA acetyltransferase cytosolic	9	8	58.5	7.76E-04	1.27
Q16658	*Fascin*	25	24	259.6	3.50E-03	1.26
P01034	*Cystatin-C*	9	8	109.4	3.78E-05	1.26
Q16719	*Kynureninase*	8	7	65.1	2.94E-03	1.26
P06396	*Gelsolin*	31	12	303.7	1.50E-02	1.26
P09382	*Galectin-1*	9	9	110.6	8.36E-03	1.24
P54578	*Ubiquitin carboxyl-terminal hydrolase 14*	2	2	10.9	8.39E-03	1.24
O00244	*Copper transport protein Atox1*	2	2	22.7	1.06E-02	1.24
P61088	*Ubiquitin-conjugating enzyme E2 N*	6	5	55.1	1.15E-03	1.24
Q15181	*Inorganic pyrophosphatase*	12	11	108.3	8.68E-04	1.23
Q16555	*Dihydropyrimidinase-related protein 2*	17	13	146.8	1.29E-02	1.20

Supplemental Table S7. Gene Ontology (GO) enrichment analysis on the 25 proteins with the highest expression in the secretome of THP-1 exposed to EVs^{PCSK9}.

GO term ID	*Description*	*FDR < 0.05*
GO:0006955	Immune response	0.00018
GO:0002376	Immune system process	0.0021
GO:0045321	Leukocyte activation	0.0056
GO:0006950	Response to stress	0.0074
GO:0030036	Actin cytoskeleton organization	0.0074
GO:0034097	Response to cytokine	0.0074
GO:0042119	Neutrophil activation	0.0074
GO:0002366	Leukocyte activation involved in immune response	0.011
GO:0010638	Positive regulation of organelle organization	0.011
GO:0050896	Response to stimulus	0.0118
GO:0007015	Actin filament organization	0.0134
GO:0002252	Immune effector process	0.0142
GO:0006952	Defense response	0.0142
GO:0043312	Neutrophil degranulation	0.017
GO:0052372	Modulation by symbiont of entry into host	0.017
GO:0071345	Cellular response to cytokine stimulus	0.017
GO:0097435	Supramolecular fiber organization	0.017
GO:0042221	Response to chemical	0.0227
GO:0007010	Cytoskeleton organization	0.024
GO:0009605	Response to external stimulus	0.024
GO:0051017	Actin filament bundle assembly	0.024
GO:0070887	Cellular response to chemical stimulus	0.0369
GO:0070301	Cellular response to hydrogen peroxide	0.0424
GO:0051707	Response to other organism	0.0433
